# A Mechanism for Amphetamine-Induced Dopamine Overload

**DOI:** 10.1371/journal.pbio.0020087

**Published:** 2004-03-16

## Abstract

xx

The notion of a neurally encoded “reward system” that reinforces pleasure-seeking behaviors first emerged fifty years ago. Psychologists James Olds and Peter Milner discovered this phenomenon when their “lack of aim” landed an electrode outside their target while studying the behavioral responses of rats given electrical stimulation to a particular brain region. It was known that stimulation of certain brain regions would induce an animal to avoid the behavior that produced the stimulus. But in the rat with the “misplaced” electrode, stimulation of this new region caused the rat to repeat the behavior that caused the stimulus. Stimulation of certain brain regions provides a very strong incentive to restimulate, creating a feedback loop that reinforces both the behavior and the neural response to it. When gentle shocks were delivered to the rat hypothalamus, for example, the animals would “self-stimulate” 2,000 times per hour by pushing a lever. The neurotransmitter dopamine, it was later discovered, plays an important role in the brain's reward system—and in laying the biochemical foundation of drug addiction.[Fig pbio-0020087-g001]

**Figure pbio-0020087-g001:**
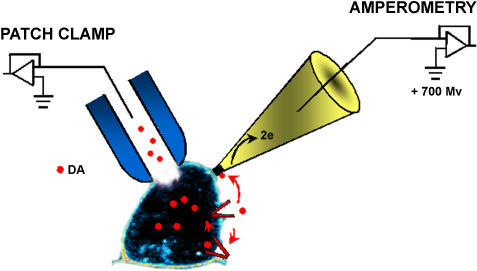
Measuring changes in dopamine transport

Essential for normal central nervous system function, dopamine signaling mediates physiological functions as diverse as movement and lactation. The dopamine transporter (DAT) is involved in terminating dopamine signaling by removing the dopamine chemical messenger molecules from nerve synapses and returning them into the releasing neurons (a process called reuptake). DAT can also bind amphetamine, cocaine, and other psychostimulants, which inhibit dopamine reuptake, and, in the case of amphetamine, also stimulate the release of dopamine through DAT. It's thought that abnormal concentrations of dopamine in synapses initiate a series of events that cause the behavioral effects of these drugs. The biochemical steps underlying amphetamine-induced dopamine release, however, are not well characterized. Now, a team led by Jonathan Javitch and Aurelio Galli has identified a chemical modification of DAT that is essential for DAT-mediated dopamine release in the presence of amphetamine. Since this modification does not inhibit the ability of DAT to accumulate dopamine, it may suggest a molecular target for treating drug addiction.

Embedded in the membrane of nerve cells, the dopamine transporter has a “tail,” called the N-terminal domain, that protrudes into the cell interior and consists of a stretch of about 60 amino acids. Many of these amino acids are potential sites of phosphorylation, a chemical reaction in which a phosphate group is added through the action of enzymes called kinases. Amphetamine has been shown to increase kinase activity and Margaret Gnegy, a coauthor of the current research article, showed previously that inhibiting protein kinase C activity blocks amphetamine's ability to release dopamine. Therefore, Javitch, Galli, and Gnegy hypothesized that N-terminal phosphorylation of DAT might play a critical role in the dopamine overload caused by amphetamine.

The researchers found that amphetamine-induced dopamine release was reduced by 80% in cells expressing a mutant dopamine transporter in which the first 22 amino acids of the N-terminal domain had been removed (del-22). Surprisingly, this truncated transporter displayed normal dopamine uptake. In a full-length DAT, mutation of the five N-terminal serine amino acids to alanine amino acids, which prevented phosphorylation, produced an effect similar to removing the 22 amino acids. In contrast, replacing these five serine residues with aspartate residues to mimic phosphorylation led to normal dopamine release as well as normal dopamine uptake.

These findings suggest that phosphorylation of one or more of these serine residues is necessary for amphetamine to flood the synapses with dopamine. While phosphorylation is a normal mechanism for regulating protein activity in a cell—and DAT is “significantly phosphorylated” under normal conditions—amphetamine could increase the level of DAT phosphorylation. Elucidating the mechanisms through which phosphorylation of DAT's N-terminus facilitates dopamine overload could lead to the development of drugs that block the “rewarding” effects of amphetamines and other addictive psychostimulants without interfering with normal dopamine clearance.

